# Effects of a physical activity intervention on schoolchildren's health-related quality of life: The active smarter kids (ASK) cluster-randomized controlled trial

**DOI:** 10.1016/j.pmedr.2018.11.002

**Published:** 2018-11-07

**Authors:** Geir Kåre Resaland, Eivind Aadland, Vegard Fusche Moe, Ronette L. Kolotkin, Sigmund A. Anderssen, John Roger Andersen

**Affiliations:** a Western Norway University of Applied Sciences, Førde, Norway; b Centre of Health Research, Førde Hospital Trust, Førde, Norway; c Western Norway University of Applied Sciences, Sogndal, Norway; d Department of Sports Medicine, Norwegian School of Sport Sciences, Oslo, Norway; e Quality of Life Consulting, Durham, NC, USA; f Duke University School of Medicine, Durham, NC, USA

**Keywords:** Physical activity, Intervention, RCT, Health-related quality of life, Kidscreen-27, Children

## Abstract

We investigated whether a seven-month (November 2014 to June 2015), school-based cluster-randomized controlled physical activity intervention improved health-related quality of life (HRQoL) in 10-year old children. The participants (*N* = 1229) from 57 elementary schools in Sogn og Fjordane County, Norway, were cluster-randomized by school either to the intervention (I) or control (C) group. The planned intervention in the 28 I-schools was 300 min of physical activity per week, compared to 135 min in the 29C-schools. HRQoL was assessed by self-report, using the Kidscreen-27 questionnaire. Objectively measured physical activity did not differ between the I–schools and C-schools during the intervention. No effect of the intervention was found for HRQoL: Physical well-being (*P* = 0.789), Psychological well-being (*P* = 0.682), Autonomy & parents (*P* = 0.662), Social support & peers (*P* = 0.828) and School environment (*P* = 0.074). In conclusion, the ASK school-based physical activity intervention showed no significant effect on HRQoL.

## 1 Introduction

Health-related quality of life (HRQoL) is a multidimensional construct encompassing physical, emotional, mental, social and behavioural components of well-being and functioning. Besides being an outcome in its own right, HRQoL in childhood is an important factor influencing personal growth and future prospects (Ravens-Sieberer, 2006).

One of the promising paths to strengthen HRQoL in childhood is to ensure an adequate level of physical activity, which might positively influence HRQoL through biological mechanisms such as reduction of stress hormones and better brain functioning, psychological ones such as excitement and mastery, and social ones such as group inclusion and belonging (Lubans et al., 2016).

However, reviews of RCTs evaluating the effects of physical activity interventions on different HRQoL outcomes in children and adolescents show a mixed picture. A review that included 25 RCTs with typically small sample sizes and 8–10 weeks of duration suggests that PA interventions are effective in improving self-esteem or self-concept in children and adolescents between 3 and 17 years, although the effect sizes often are small (Liu et al., 2015). On the other hand, a review of 11 RCTs with large-scale school based interventions, with typically 1 year of follow-up; suggests none or only trivial effects on wellbeing in children and adolescents between 4 and 16 years, even in the presence of statistically modest significant increases in objectively measured physical activity (Rafferty et al., 2016). To our best knowledge, only 4 out of 11 studies included in the review of Rafferty et al., used measures that were broad enough to capture the multidimensional nature of HRQoL (i.e., including physical, emotional and social domains). Surprisingly, in two of the three studies that showed an effect on HRQoL outcomes in the latter review, no significant increases in physical activity were shown in the intervention groups.

In conclusion, it is hard to draw any firm conclusion based on the current knowledge base. However, it is possible that physical activity interventions that include many schools and have longer follow up induce more complexity than smaller studies. Consequently, the positive effect seen in in the review on self-esteem or self-concept might reflect that these studies are simpler to conduct as planned. It has also been argued that the research in this field has been hampered, as outcomes often have not captured the multidimensional nature of wellbeing from the child point of view (Rafferty et al., 2016).

The aim of this paper was to evaluate the effects of a seven-month school-based physical activity intervention on HRQoL in a large sample of 10-year old children across 57 schools. We also studied the moderating effect of gender and baseline HRQoL on participation in the intervention and HRQoL.

## 2 Methods

The present paper used data from the ASK study—a cluster-randomized controlled trial conducted in Sogn og Fjordane County, Norway, between August 2014 and June 2015. We have previously published (open access) a detailed description of the ASK study and its main effects (Resaland et al., 2016; Resaland et al., 2015). Therefore, only a brief description of the study is provided here. The principal aim of the ASK-study was to study the effects of a physical activity intervention on academic performance in 10-year-old children. No overall difference in objectively measured physical activity between the intervention and control group were found during the intervention (Resaland et al., 2016). However, the study also suggest that combining physical activity and learning may be a viable model for better learning in the children with weaker academically performance. HRQoL was defined as a secondary outcome in the ASK-study protocol.

We randomized 28 schools (596 children) to the intervention group (I-schools), and 29 schools (533 children) to the control group (C-schools). The study was powered to detect an effect size of 0.35 between two groups for change in the primary outcome (academic performance). We obtained written consent from the parents or legal guardians and responsible school authorities of each child. The Regional Committee for Medical Research Ethics approved the trail (reference number: 2013/1893). Clinicaltrials.gov ID number: NCT02132494 (Aadland, 2014).

### 2.1 Intervention

The intervention in the I-schools was 165 min extra time of teacher-led physical activity per week: physically active lessons conducted in the playground (90 min/week), physical activity breaks during academic lessons (25 min/week), and physical activity homework prepared by teachers (50 min/week). In addition, both I-schools and C-schools participated equally in the curriculum-prescribed physical activity (135 min/week).

### 2.2 Measures

The primary outcome on this paper is child reported HRQoL, assessed with the Kidscreen-27 questionnaire, which has five domains: physical well-being; psychological well-being; autonomy & parents; social support & peers; and school environment, and have good psychometric properties (Andersen et al., 2016; Ravens-Sieberer, 2006). This questionnaire was designed to capture the multidimensional nature of HRQoL. Higher scores indicate a better HRQoL. Kidscreen-27 norm scores in girls and boys combined in the age group 8–11 years are: physical well-being (mean = 53.7); psychological well-being (mean = 50.1); autonomy & parents (mean = 51.6); social support & peers (mean = 52.0); and school environment (mean = 54.0) (Ravens-Sieberer, 2006). The children completed the Kidscreen-27 while seated at their desks in the classroom. The teachers instructed the children to carry on with their schoolwork until all children were finished. The children could ask the teacher for help if they did not understand something.

Physical activity was measured using triaxial accelerometers (ActiGraph GT3X+, LLC, Pensacola, Florida, USA) over seven consecutive days before the intervention started and at the end of the trail period. The outcomes applied in this study were total physical activity per day and during the school day (09:00–14:00) defined as counts per minute (CPM). Waist circumference was measured with an ergonomic circumference measuring tape.

### 2.3 Statistics

Means and standard deviations (SD) were used to describe continuous variables, and absolute numbers and percentages for binary variables. The intervention effect was analysed using an intention-to-treat analysis. Missing data were imputed from relevant variables by means of multiple imputations using a Markov Chain Monte Carlo procedure with 20 iterations. We assumed data were missing at random. The main analysis was conducted using five separate linear mixed-effect models with the post scores for the five Kidscreen-27 domains as outcomes. The independent variable was I-schools versus C-schools (binary predictor) adjusted for the baseline Kidscreen-27 score. School site was included as a random effect. We also conduced secondary interactions analyses (gender ∗ treatment group and baseline HRQoL ∗ treatment group) to test for moderating effects of gender and baseline scores on HRQOL.

We report group differences (unstandardized regression coefficients) and their 95% confidence intervals. Effect sizes (ES) were calculated by dividing the adjusted group difference in HRQOL between I-schools versus C-schools by a value of 10, which is the standard deviation of the Kidscreen-27 scores across Europe. The developers of the Kidscreen-27 have recommend using the following standard criteria interpretation: trivial (<0.2 ES), small (0.2–0.5 ES), moderate (0.5–0.8 ES) and large (>0.8 ES) (Ravens-Sieberer et al., 2007). These cut-offs are in agreement with findings from a range of populations (Fuller et al., 2004). A two-sided *P* value <0.05 indicated statistical significance. No adjustment for multiple testing was performed (Perneger, 1998). Statistical analyses were performed using IBM SPSS, version 23.0 for Windows.

## 3 Results

**Fig. 1:**
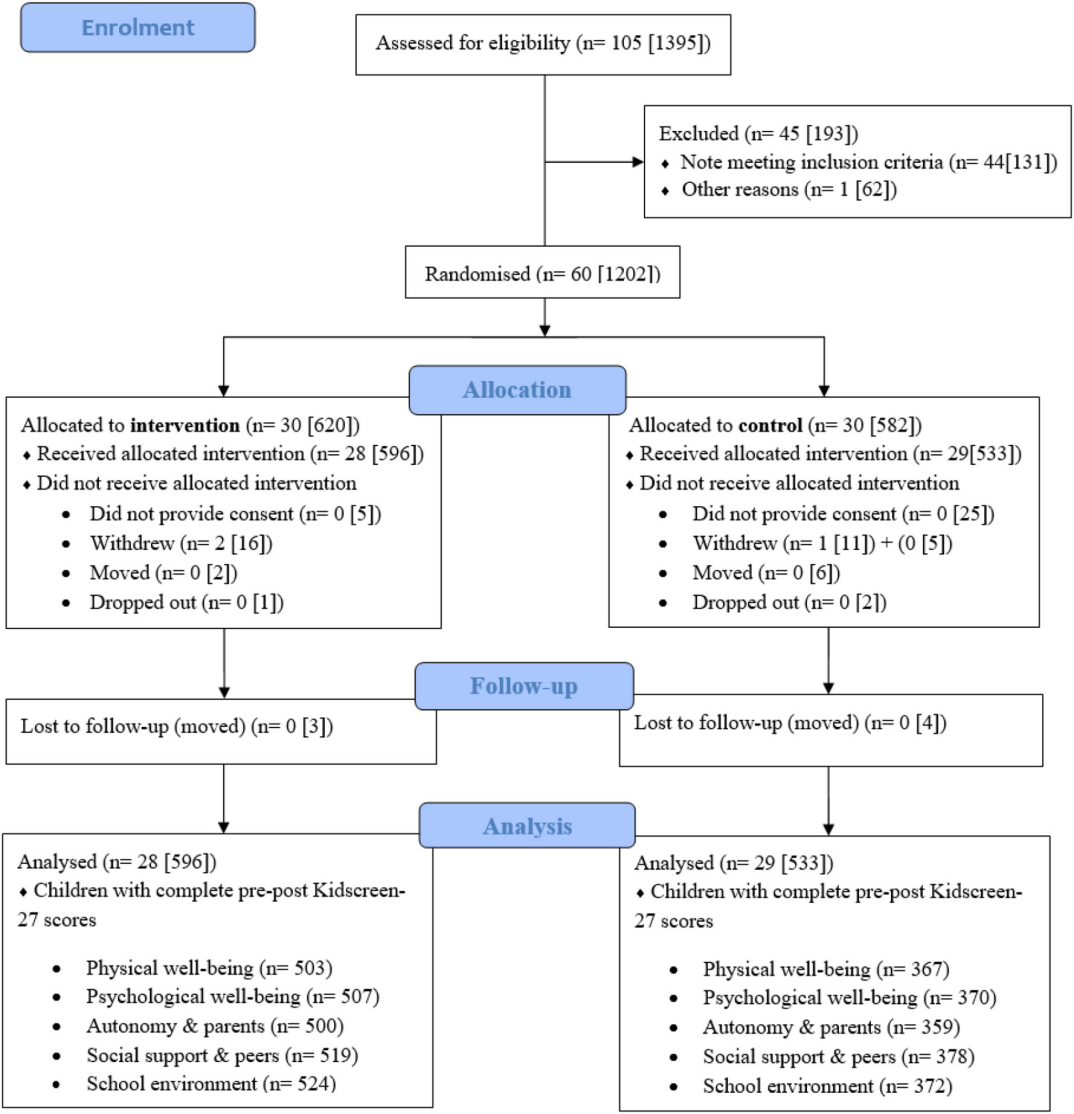
The consort flow diagram. Flow of schools and children through the study. All numbers are schools [children].

The participant's characteristics and study flow are presented in Table 1 and Fig. 1. We found no effect of the intervention on any HRQoL domains (Table 2). Furthermore, no significant interactions effects were found for gender ∗ treatment group (*P*-values from 0.089 to 0.164), or for baseline HRQoL ∗ treatment group (*P*-values from 0.105 to 0.494) in relation to the five HRQoL post scores. Regarding unintended harmful effects of the study, we observed none.

**Table 1:** Study participants baseline characteristics (*N* = 1129).

Variables	Intervention (*n* = 596)	Control (*n* = 533)
Age (years), mean ± sd	10.2 ± 0.3	10.2 ± 0.3
Gender (girls), (%)	47.3	48.6
Waist circumference, mean ± sd	61.8 ± 7.5	62.4 ± 7.6
Physical activity, mean ± sd		
Counts per minute (all day)	739 ± 298	720 ± 264
Counts per minute (at school)	650 ± 185	641 ± 192
Kidscreen-27, mean ± sd		
Physical well-being	51.2 ± 10.5	50.7 ± 10.1
Psychological well-being	52.4 ± 9.7	52.4 ± 9.3
Autonomy & parents	49.9 ± 9.7	50.3 ± 9.5
Social support & peers	50.2 ± 9.8	51.4 ± 9.6
School environment	52.9 ± 9.9	53.8 ± 9.6

**Table 2:** Adjusted Kidscreen-27 follow-up scores, and group differences, means, 95% CIs, *P*-values and effect sizes.

Domain	Intervention Mean (95% CI)	Control Mean (95% CI)	Group difference Unstandardized reg. coefficient (95% CI)	ES
Physical well-being	51.3 (50.2, 53.4)	51.5 (50.4, 52.6)	−0.2 (−1.8, 1.4); *P* = 0.789	0.0
Psychological well-being	53.5 (52.5, 54.4)	53.8 (52.8, 54.8)	−0.3 (−1.7, 1.1); *P* = 0.682	0.0
Autonomy & parents	52.3 (51.4, 53.2)	52.0 (51.0, 53.0)	0.3 (−1.1, 1.7); *P* = 0.662	0.0
Social support & peers	51.2 (50.2, 51.1)	51.0 (50.0, 52.0)	0.2 (−1.2, 1.5); *P* = 0.828	0.0
School environment	54.3 (53.5, 55.2)	55.3 (54.4, 56.3)	−1.0 (−2.3, 0.2); *P* = 0.074	−0.1

Note: the follow-up scores were adjusted for the corresponding baseline Kidscreen-27 value and school site using mixed effect modelling. ES: effect size, calculated by dividing the group adjusted difference in HRQoL between I-schools versus C-schools by a value of 10, which is the standard deviation of the Kidscreen-27 scores across Europe.

## 4 Discussion

We found no significant effect of the ASK school-based physical activity intervention on HRQoL. Strengths of the study are the cluster RCT design, large sample size, and valid measures of physical activity and HRQoL. The major limitation is the non-significant difference in physical activity between the intervention and control group during the intervention. Consequently, how sensitive the Kidscreen-27 is for change related to physical activity in studies like the present one remains to be documented. We planned to implement 165 min extra minutes of teacher lead physical activity per week (33 min per day), aiming for a total of 60 min/day including the mandatory education. Although, the teachers in the I-schools reported having conduced the pre-defined interventions, this was not reflected in an increase in objectively measured physical activity. The differences between reported and objectively measured physical activity levels might reflect a reporting bias by the teachers, but could also result from the limited ability of accelerometers to capture some types of physical activity. It is also possible that the teachers carried out the intervention as requested while the child's physical activity intensity remained the same.

Our findings are in agreement with other large school-based physical activity interventions in children, which also have demonstrated little or no effect on HRQoL (Rafferty et al., 2016). A possible explanation is that exposure in the intervention groups generally have been insufficient. Furthermore, there might be a lag effect before a physical activity intervention leads to changes in HRQoL. For example, it might take time until new physical activity related learning strategies are mastered by teachers and pupils.

The children's Kidscreen-27 scores were in the normal range compared to European population norms, and the floor effects at baseline ranged from 0% to 0.3% while ceiling effects ranged from 6.3% to 17.1% (Andersen et al., 2016). This suggests that there is potential for change for the majority of participants. Thus, an interesting issue is how much change in physical activity is needed to influence HRQoL in relatively healthy children. This is not well established, but cross-sectional data based on the present sample have suggested that as much as 1.2 SD of extra physical activity (331 counts per minute, all day) might be needed to improve the Kidscreen-27 domain Physical well-being by 0.2 SD (Andersen et al., 2017). Thus, future studies should have high statistical power and a strict exposure control as prioritised design elements. On the other hand, the quality of the physical activity intervention, rather than the quantity, might also play a role. Thus, whether different physical activity related learning activities differ in their effect on HRQoL is a topic for future research.

## 5 Conclusion

In conclusion, we did not find any effect on HRQoL in this RCT. In their review of this field, Rafferty et al. (Rafferty et al., 2016), have concluded that the effects of school based physical activity interventions on HRQoL seem to be more complex that originally believed. Our paper add to the body of knowledge, and may help point the way to further research that might result in a greater understanding of this complexity.

## Abbreviations

ASK: active smarter kids
Cluster RCT: cluster randomized controlled trial
I-schools: Intervention schools
C-schools: control schools
HRQoL: health-related quality of life
CPM: count per minute
ES: effect size

## Competing interests

The author declares no conflict of interest.

## Funding

This study was funded by the Research Council of Norway (ID number 221047/F40) and Western Norway University of Applied Sciences.
